# Optimizing the Performance of CsPbI_3_-Based Perovskite Solar Cells via Doping a ZnO Electron Transport Layer Coupled with Interface Engineering

**DOI:** 10.1007/s40820-019-0320-y

**Published:** 2019-10-18

**Authors:** Man Yue, Jie Su, Peng Zhao, Zhenhua Lin, Jincheng Zhang, Jingjing Chang, Yue Hao

**Affiliations:** State Key Discipline Laboratory of Wide Band Gap Semiconductor Technology, Shaanxi Joint Key Laboratory of Graphene, School of Microelectronics, Xidian University, 2 South Taibai Road, Xi’an, 710071 People’s Republic of China

**Keywords:** All-inorganic CsPbI_3_ perovskites, Interface engineering, Doping, ZnO, Simulation

## Abstract

Interface engineering has been regarded as an effective and noninvasive means to optimize the performance of perovskite solar cells (PSCs). Here, doping engineering of a ZnO electron transport layer (ETL) and CsPbI_3_/ZnO interface engineering via introduction of an interfacial layer are employed to improve the performances of CsPbI_3_-based PSCs. The results show that when introducing a TiO_2_ buffer layer while increasing the ZnO layer doping concentration, the open-circuit voltage, power conversion efficiency, and fill factor of the CsPbI_3_-based PSCs can be improved to 1.31 V, 21.06%, and 74.07%, respectively, which are superior to those of PSCs only modified by the TiO_2_ buffer layer or high-concentration doping of ZnO layer. On the one hand, the buffer layer relieves the band bending and structural disorder of CsPbI_3_. On the other hand, the increased doping concentration of the ZnO layer improves the conductivity of the TiO_2_/ZnO bilayer ETL because of the strong interaction between the TiO_2_ and ZnO layers. However, such phenomena are not observed for those of a PCBM/ZnO bilayer ETL because of the weak interlayer interaction of the PCBM/ZnO interface. These results provide a comprehensive understanding of the CsPbI_3_/ZnO interface and suggest a guideline to design high-performance PSCs.

**Electronic supplementary material:**

The online version of this article (10.1007/s40820-019-0320-y) contains supplementary material, which is available to authorized users.

## Highlights

- Device simulations and first-principle calculations are employed to derive a guideline for the optimization of CsPbI_3_-based perovskite solar cells (PSCs).
- The open voltage and power conversion efficiency of the PSCs are, respectively, improved to 1.31 V and 21.06% by simultaneously introducing an ultra-thin TiO_2_ buffer layer and increasing the doping concentration of the ZnO electron transport layer.
- The influence of the interfacial buffer layer and doping of the CsPbI_3_/ZnO interface on PSC performance is discussed.

**Electronic supplementary material:**

The online version of this article (10.1007/s40820-019-0320-y) contains supplementary material, which is available to authorized users.

## Graphical abstract

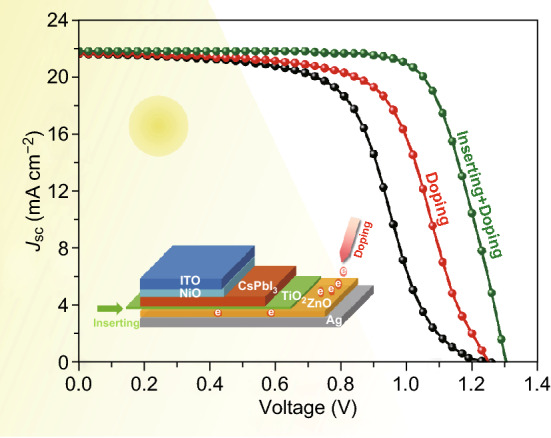

## Introduction

Organolead halide perovskites (OHPs) have been regarded as promising absorber materials for photovoltaic devices owing to their excellent physical and fabrication properties, such as high absorption coefficients, long charge carrier diffusion lengths, and roll-to-roll processing approaches [1–7]. The record power conversion efficiency (PCE) of OHP solar cells (PSCs) has also increased from 3.8 to 25.2% within the last few years [8]. However, the application of such solar cells is limited by the stability of organic cations given their hygroscopic and volatile nature [9, 10]. To overcome these issues, several theoretical and experimental investigations have been completed to fabricate all-inorganic CsPbX_3_ perovskites given the instability of PSC with organic cations [11–16]. Moreover, CsPbX_3_ can sustain temperatures exceeding 400 °C without any phase degradation [17, 18]. CsPbBr_3_, CsPbI_3_, CsPbI_2_Br, and CsPbIBr_2_ are the most studied all-inorganic perovskites for photovoltaic application [19–22]. Notably, CsPbBr_3_, CsPbI_2_Br, and CsPbIBr_2_ possess large bandgaps that are not ideal or appropriate for a solar cell [23, 24], while CsPbI_3_ shows a more suitable bandgap of 1.73 eV for photovoltaic application, particularly for a double-junction perovskite/Si tandem [4, 23, 25–27]. Moreover, the PCE of CsPbI_3_-based PSCs has shown a reproducible photovoltaic performance with a champion efficiency up to 17% [28]. Nevertheless, such reported performances of CsPbI_3_-based PSCs remain lower than expected.

As is known, the performance of PSCs is not only influenced by the perovskite itself but also strongly affected by the interface between the perovskite and electron transport layer (ETL) which directly affects the collection efficiency of photo-induced charge carriers and PSC stability. To obtain excellent performances of PSCs, several ETLs such as those of titanium oxide (TiO_2_), zinc oxide (ZnO), tin dioxide (SnO_2_), and n-type organic molecules have been attempted [29–34]. Among them, ZnO has been widely investigated because of its direct wide bandgap, ultra-high electron mobility, transparent properties, and ease of processing at low temperature [30, 35, 36]. Moreover, the ZnO ETL has promoted the open-circuit voltage (*V*_oc_) and PCE of CH_3_NH_3_PbI_3_-based PSCs to an exciting level (*V*_oc_ > 1.20 V and PCE > 21%) [36, 37]. Nevertheless, regarding the all-inorganic perovskite-based PSCs with a ZnO ETL, most of their superior performances have yet to be fully realized and few have been studied in practical applications. For example, CsPbI_2_Br-based PSCs with a ZnO ETL show a low PCE of approximately 13% accompanying a low extraction efficiency and a severe charge recombination [38, 39]. Previous reports regarding OHP-based PSCs have showed that inserting a buffer layer between the perovskite and ETL is an effective means to optimize the performances of OHP-based PSCs [36, 40]. Inspired by these, Jeong et al. [41] inserted poly(ethylene oxide) between the CsPbI_3_ and ZnO ETL to enhance the phase stabilization of α-CsPbI_3_. Our previous studies have employed an MoO_3_ interfacial layer to enhance charge extraction and suppress carrier recombination of a CsPbI_2_Br-based PSC [13]. Nevertheless, the performances of CsPbI_3_-based PSCs remain lower than expected. The influential mechanisms of these methods on all-inorganic CsPbI_3_ devices have not yet been comprehensively investigated. To further understand the effects of inserting a layer, the CsPbI_3_-based PSCs with a ZnO ETL coupling with ultra-thin PCBM and TiO_2_ inserting layers are designed and investigated by device simulations and first-principle calculations. In addition, previous studies have showed that doping the ETL is another means to tune the performances of PSCs. Metal ion (e.g., Mg, Li, Al, and Nb)-doped ZnO has been used as an efficient ETL to enhance the PCE to greater than 19% [42–44]. Moreover, novel molecular (e.g., triphenylphosphine oxide (TPPO) and phenyl-C61-butyric acid methyl ester (PCBM)) doping of the ETL has relieved the current hysteresis and increased the PCE of an OHP-based PSC from 19.01 to 20.69%. Inspired by these results, modulating the doping concentration in the ZnO layer of a CsPbI_3_-based PSC with PCBM and TiO_2_ inserting layers is employed to further improve the performances of CsPbI_3_-based PSCs in this study.

Herein, the device simulations implemented in the Silvaco technology computer-aided design (TCAD) simulation code and the first-principle calculations implemented in the Vienna Ab initio simulation package (VASP) codes are employed to investigate the CsPbI_3_/ZnO interface of CsPbI_3_-based PSCs. The energy bands, photo-generation rate, current density–voltage (*J*–*V*) characteristics, and spectral response can be calculated by Silvaco TCAD. The interfacial properties based on electronic and atomic structures can be showed by the first-principle calculations. Consequently, the optimal doping concentration of ZnO ETL and the thicknesses of the TiO_2_ and PCBM insertion layers are obtained. At the same time, the effects and mechanisms of the doping concentration and insertion layers on the PCE, *V*_oc_, fill factor (FF), and current tailing phenomenon are analyzed by energy bands, photo-generation rate, interfacial structures, and density of states (DOSs). Moreover, the 1-nm ultra-thin TiO_2_ insertion layer coupling with the doped ZnO layer at a 10^22^ cm^−3^ doping concentration can improve the *V*_oc_ and PCE of the CsPbI_3_-based PSCs from 1.25 V and 15.09% to 1.31 V and 21.06%, respectively. Our work can provide important guidance and understanding for device design and optimization from the considerations of theory.

## Simulation Methods

All device simulations were conducted using Silvaco TCAD which was mainly based on the Poisson equation (Eq. 1), carrier continuity equation (Eq. 2), and drift–diffusion equation (Eq. 3) as follows [4, 31]:

1 $$\frac{{a^{2} \varphi }}{{ax^{2} }} = \frac{q}{\varepsilon }(n - p)$$

2 $$\frac{an}{at} = \frac{1}{q}\frac{{aJ_{n} }}{ax} + G - R\frac{ap}{at} = - \frac{1}{q}\frac{{aJ_{p} }}{ax} + G - R$$

3 $$J_{n} = qD_{n} \frac{{a_{n} }}{ax} - q\mu_{n} \frac{{a_{\varphi } }}{ax}J_{p} = - qD_{p} \frac{{a_{p} }}{ax} - q\mu_{p} \frac{{a_{\varphi } }}{ax}$$

where $J_{n}$ is the electron current density, $J_{p}$ is the hole current density, $D_{n}$ is the electron diffusion coefficient, $D_{p}$ is the hole diffusion coefficient, $\mu_{n}$ is the electron mobility, $\mu_{p}$ is the hole mobility, $\varphi$ is the electric potential, $\varepsilon$ is the dielectric constant, *q* is the electron charge, *n* is the electron concentration, *p* is the hole concentration, *G* is the carrier generation rate, and *R* is carrier recombination rate. In the simulation, Shockley–Read–Hall (SRH), band-to-band, and Auger recombinations were considered. The transfer-matrix method (TMM) was used as an optical model to calculate the carrier generation rate *G*(*x*) [45]. According to Eqs. (4) and (5), the optical electric field |*E*(*x*)|^2^ should be obtained before calculating the carrier generation rate. Here, *ε*_0_, *c*, *k*, *n*, *h*, and *λ* are the vacuum permittivity, light speed, imaginary part of the refractive index, real part of the refractive index, Planck constant, and wavelength, respectively:

4 $$Q(x,\lambda ) = \frac{{2\pi c\varepsilon_{0} kn|E(x)|^{2} }}{\lambda }$$

5 $$G\left( x \right) = \mathop \int \limits_{{\lambda_{1} }}^{{\lambda_{2} }} \frac{\lambda }{hc}Q(x,\lambda ){\text{d}}\lambda .$$

In addition, the standard AM 1.5 G solar spectrum was used to measure the *J*–*V* curve under illumination. Such approaches have been widely employed to investigate the optical behaviors of photovoltaics based on perovskites [4, 5, 31, 46–48].

**Fig. 1:**
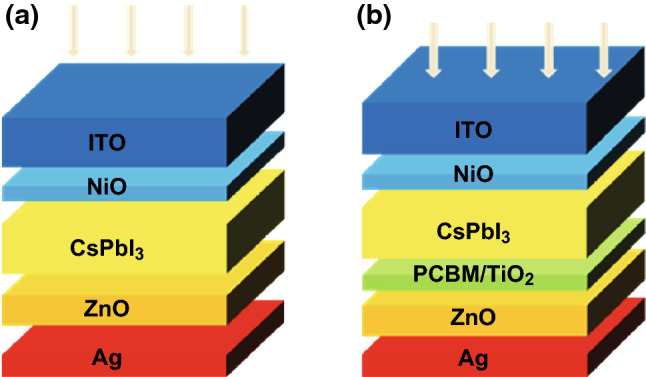
Schematic device structure of the CsPbI_3_-based PSCs with **a** ZnO single-layer and **b** TiO_2_ (PCBM)/ZnO bilayer ETL

The inverted PSCs based on CsPbI_3_ in this study are shown in Fig. 1. CsPbI_3_ was employed as an absorber layer and NiO as a hole transport layer. The electron transport layers included a ZnO single layer, TiO_2_/ZnO, and PCBM/ZnO bilayer, respectively. During the experiment, the similar structure device Au/NiO/CsPbBr3/ZnO/ITO/glass was successfully prepared [34]. To comprehensively understand the mechanism of the ETL and eliminate the effect of the hole transport layer, the optimal thickness of the NiO hole transport layer was set to 30 nm and was unchanged after an extensive test as shown in Fig. S1.

All first-principle density functional theory calculations were performed using the projector augmented wave method (PAW) and Perdew–Burke–Ernzerhof (PBE) implemented in the VASP code [49–51]. The convergence criterions were 1 × 10^−5^ eV for the self-consistent field energy and 0.01 eV Å^−1^ for the residual forces on each atom, respectively. A cutoff energy of 400 eV and a *k*-point sampling at the gamma point were employed. The CsPbI_3_/ZnO interface models were constructed using a 1 × 1 supercell of the CsPbI_3_ (001) surface and a 2 × 2 supercell of the ZnO (001) surface. A vacuum of 15 Å was considered along the z direction to avoid artificial interlayer interactions.

## Results and Discussion

**Fig. 2:**
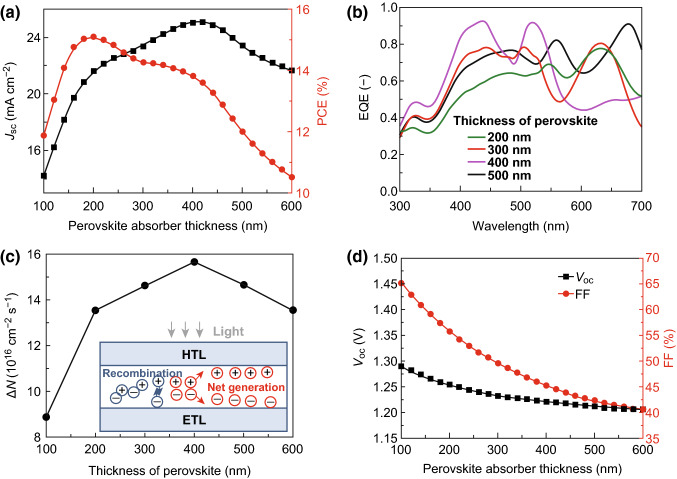
**a**, **d** Device parameters as functions of the perovskite thickness for a CsPbI_3_-based PSC with a single-layer ZnO ETL. **b** EQE spectra and **c** net carrier generation rate for CsPbI_3_-based PSCs with a single-layer ZnO ETL under different perovskite thickness conditions

**Fig. 3:**
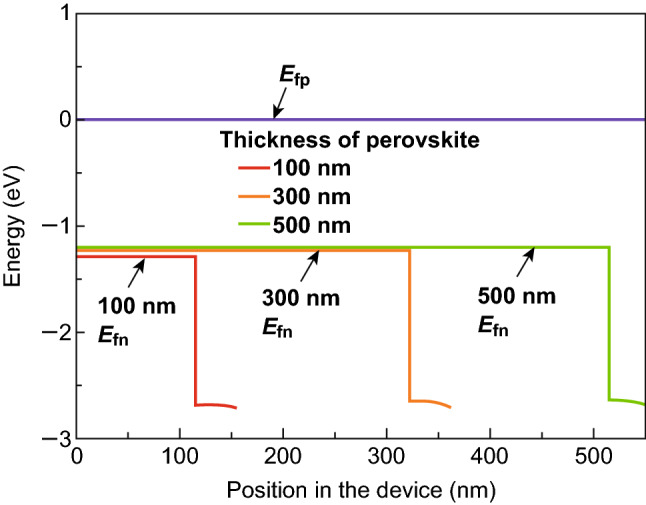
Quasi-Fermi level with different perovskite layer thicknesses

According to previous studies, both the thicknesses of the absorber layer and electron transport layer strongly affect the performance of a solar cell device; thus, it is necessary to optimize the thicknesses of the perovskite and ZnO ETL. Figure 2a shows the short-circuit current density (*J*_sc_) for the CsPbI_3_-based PSCs with a single-layer ZnO ETL depending on the thickness of the CsPbI_3_. The electrical parameters of such PSCs are summarized in Table 1 [23, 24, 31, 52, 53]. *J*_sc_ sharply increases at first and then decreases with increasing perovskite thickness. The maximum *J*_sc_ reaches 25.05 mA cm^−2^ when the perovskite thickness reaches 420 nm. This can be attributed to the variation in the external quantum efficiency (EQE) and net carrier generation rate (*ΔN*) of the PSCs as shown in Fig. 2b, c. It can be seen that the EQE of the PSCs is enhanced with the increasing thickness of the CsPbI_3_, suggesting the stronger photo-absorption and higher carrier generation rate. Meanwhile, the carrier diffusion length of CsPbI_3_ of approximately 1.5 μm [54] is longer than its thickness. This might result in increasingly more generated carriers being collected by electrodes with negligible recombination. As a consequence, the *ΔN*s (the carrier generation rate subtracted by the carrier recombination rate) of the PSCs shifts up accompanying the enhanced *J*_sc_, as shown in Fig. 2c. When the CsPbI_3_ thickness continues to increase and exceeds 420 nm, the EQE of the PSC decreases and this in turn decreases the carrier generation rate. Meanwhile, the enhanced thickness can enlarge the recombination rate of the generated carriers. As a result, the less generated carries are collected by electrodes, leading to the reduced net carrier generation rates of the PSCs accompanying the decreasing *J*_sc_. For the PCE of the CsPbI_3_-based PSCs, its variation is similar to that of *J*_sc_ except for the thickness corresponding to the maximum PCE, as shown in Fig. 2a. The maximum PCE of the CsPbI_3_-based PSC with a single-layer ZnO ETL is 15.09%, and the corresponding thickness of the CsPbI_3_ perovskite is approximately 200 nm rather than 420 nm. Thus, 200-nm CsPbI_3_ is employed in the following discussion. Notably, such characteristics are different from those of OHP-based PSCs [4, 31, 46, 55] because both the *V*_oc_ and FF monotonously decrease as the thickness of the CsPbI_3_ increases from 100 to 600 nm, which is different from that of *J*_sc_, as shown in Fig. 2d and previous reports regarding OHP-based PSCs [4, 31]. To elucidate such variation, Fig. 3 shows the quasi-Fermi level with different perovskite layer thicknesses because the difference between the quasi-Fermi levels directly affects the energy required for carrier transition. It is obvious that the difference between the electron quasi-Fermi level (*E*_fn_) and hole quasi-Fermi level (*E*_fh_) decreases with the increase in the perovskite layer thickness, which corresponds with the continuously decreased energy required for carrier transition. Thus, the *V*_oc_ monotonously decreases as the CsPbI_3_ thickness increases.

**Table 1:** Simulation parameters of the perovskite solar cell, where $\varepsilon_{r}$ is the dielectric constant, *E*_g_ is the bandgap, *λ* is the electron affinity, *N*_c_ is the effective conduction band density, *N*_v_ is the effective valence band density, $\mu_{n}$ is the electron mobility, and $\mu_{p}$ is the hole mobility

Parameters	NiO	CsPbI_3_	PCBM	ZnO	TiO_2_
Thickness (nm)	30	200	8	40	1
$\varepsilon_{r}$	12	6	4	9	100
*E*_g_ (eV)	3.6	1.73	2	3.3	3.2
*λ* (eV)	1.7	3.6	3.9	4.4	4
*N*_C_ (cm^−3^)	2.5 × 10^20^	1.49 × 10^18^	1 × 10^21^	2.2 × 10^18^	1 × 10^21^
*N*_V_ (cm^−3^)	2.5 × 10^20^	2.2 × 10^18^	2 × 10^20^	1.8 × 10^19^	2 × 10^20^
*N*_A_ (cm^−3^)	1 × 10^16^	–	–	–	–
*N*_D_ (cm^−3^)	–		1 × 10^20^	1 × 10^20^	1 × 10^20^
$\mu_{n}$ (cm^2^ V^−1^ s^−1^)	0.01	25	0.01	100	0.006
$\mu_{p}$ (cm^2^ V^−1^ s^−1^)	0.01	25	0.01	25	0.006

**Fig. 4:**
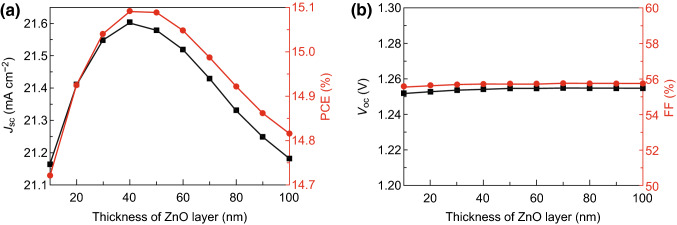
Device parameters as a function of the thickness of ZnO for the CsPbI_3_-based PSC with a single-layer ZnO ETL

Figure 4 shows the performance of CsPbI_3_-based PSCs with a single-layer ZnO ETL as a function of the ZnO thickness. Here, the CsPbI_3_ thickness is set to 200 nm. It was found that the *J*_sc_ increases as the ZnO layer thickness increases from 10 to 40 nm and then decreases with the continued increasing thickness of the ZnO layer. The EQE of the PSC, which directly determines the numbers of generated carriers, first increases and then decreases with the increasing thickness of the ZnO layer, as shown in Fig. S2a. In other words, as the ZnO layer thickness continues to increase, the net carrier generation rate of the PSC cannot monotonously increase but reaches a peak value immediately when the ZnO layer thickness is 40 nm, as shown in Fig. S2a. As a result, *J*_sc_ shows its highest value of approximately 21.61 mA cm^−2^ when the ZnO layer thickness is 40 nm. Such similar variation characteristics are also suitable to the change in the PCE with the increasing thickness of the ZnO layer because the *V*_oc_ and FF remain unchanged irrespective of the thickness of the single-layer ZnO (Fig. 4b). This is because the net carrier generation rate has not influenced the band energy and interfacial structure of the CsPbI_3_/ZnO interface which are the intrinsic properties of the fabricated PSC. The differences between the band edges of the perovskite and ZnO layers that directly affect the *V*_oc_ and the defect states density that affects the FF remain unchanged even if the thickness of the ZnO layer increases, as shown in Fig. S2b. Thus, the optimal thickness of the ZnO ETL is 40 nm and the optimized PCE is approximately 15.09%.

**Fig. 5:**
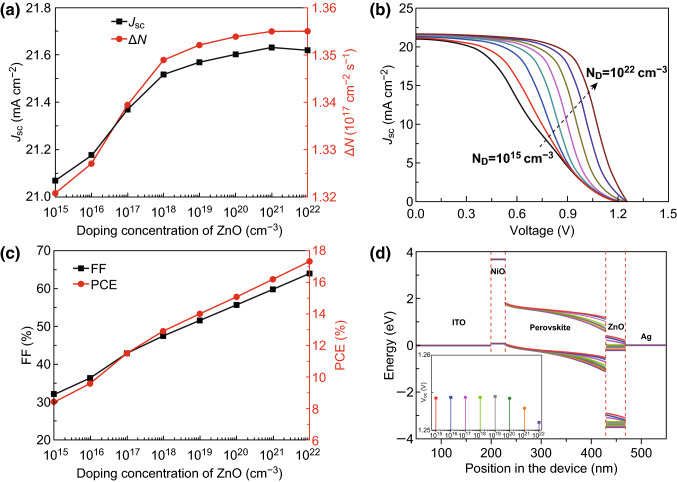
**a** Short-circuit current density (*J*_sc_) and net carrier generation rate (*ΔN*), **b** *J*–*V* curves, **c** fill factor (FF) and power conversion efficiency (PCE), and **d** energy band diagrams coupled with open-circuit voltage *V*_oc_ of CsPbI_3_-based PSCs at different doping concentrations

In addition, notably, although the *V*_oc_ is sufficiently large (approximately 1.254 V), the PCE and FF are far lower than those of the OHP-based PSCs [46]. An obvious current trailing in the *J*–*V* curve, which is related to the conductivity of ETL, is observed for the CsPbI_3_-based PSCs with a single-layer ZnO ETL, as shown in Fig. S3. Thus, doping of an ETL, such as an in-doped ZnO ETL, has been employed to improve the performance of the PSCs during experiments [56]. Nevertheless, the optimal doping concentration and doping mechanism have not yet been determined. Figure 5a shows the *J*_sc_ of a CsPbI_3_-based PSC with a different doping concentration in the single-layer ZnO ETL. It was found that the *J*_sc_ shifts up at first as the doping concentration increases and then reaches a maximum value of approximately 21.62 mA cm^−2^ at a doping concentration of approximately 10^21^ cm^−3^ before and finally shifting down as the doping concentration continues to increase. However, the net carrier generation rates first increase and then approach a constant with increasing doping concentration. On the one hand, the dopant can improve the charge density (Fig. S4) and then improve the conductivity of the ZnO layer such that the recombination rate of the photo-generated carrier around the interface decreases. On the other hand, the doping concentration in the single-layer ZnO ETL has a negligible influence on the EQE (Fig. S5) such that the number of photo-generated carriers remains unchanged. As a result, the dopant increases the net carrier generation rate (Fig. 5a) and then improves the short-circuit current density. Note that although the dopant improved the photo-generated carrier density, the dopant inevitably induces impurity scattering, which can deteriorate the carrier mobility. When the doping concentration is sufficiently large, the negative effect of impurity scattering can outweigh the positive effect of the dopant, leading to a stronger reduction in the carrier mobility than the increment of the carrier density. Meanwhile, the highest carrier recombination rate of a semiconductor is closely related to the temperature and independent of the carrier concentration [57]. Consequently, the short-circuit current density decreases, and the net carrier generation rate remains constant when the doping concentration of the ZnO layer is larger than 10^21^ cm^−3^. In addition, because of the enhanced conductivity via the dopant, less carriers are accumulated in the CsPbI_3_/ZnO contact region, which relieves the obvious current trailing in the *J*–*V* curve, as shown in Fig. 5b. Owing to the reduced current trailing, both the FF and PCE of the CsPbI_3_-based PSC with a single-layer ZnO ETL monotonically increase with increasing doping concentration (in Fig. 5c). Nevertheless, it is interesting that the *V*_oc_ remains at a value of 1.254 V as the doping concentration increases and then slightly decreases when the doping concentration in the single-layer ZnO ETL is higher than 10^20^ cm^−3^. Although both the band edges of the CsPbI_3_ and ZnO at the CsPbI_3_/ZnO contact region continually shift down with increasing doping concentration, the difference between the conduction band maximum (CBM) of the CsPbI_3_ and valence band minimum (VBM) of the ZnO remains constant, leading to an unchanged built-in electric field at the CsPbI_3_/ZnO contact. Furthermore, when the doping concentration in the single-layer ZnO ETL is greater than 10^20^ cm^−3^, the reduced CBM of the ZnO ETL is lower than the Fermi level of the electrode, which can induce an opposite electric field at the ZnO/Ag contact and then offset the partial electric field at the CsPbI_3_/ZnO contact. Thus, the *V*_oc_ slightly decreases when the doping concentration in the single-layer ZnO ETL is greater than 10^20^ cm^−3^, as shown in the inset of Fig. 5d.

**Fig. 6:**
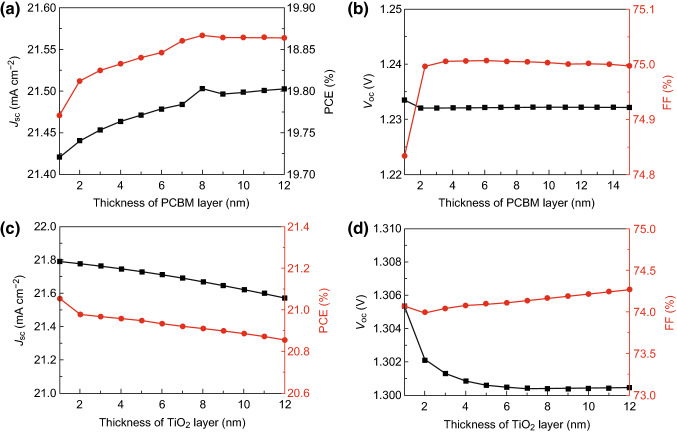
Device parameters as functions of **a**, **b** the thickness of the PCBM layer for a PSC with a PCBM/ZnO bilayer ETL and **c**, **d** the thickness of the TiO_2_ layer for a PSC with TiO_2_/ZnO bilayer ETL

Except for doping of the ETL, inserting an additional ultra-thin buffer layer between the perovskite and ETL to form a bilayer ETL has also been employed to improve the performance of PSCs [58]. Here, PCBM and TiO_2_ are selected as the additional buffer layers and combined with the ZnO electron transport layer to form PCBM/ZnO and TiO_2_/ZnO bilayer ETLs (as shown in Fig. 1). Meanwhile, the ZnO doping concentration is set to 10^20^ cm^−3^. To exclude the effect of CsPbI_3_ thickness, the thicknesses of all CsPbI_3_ parts are set to 200 nm. For the CsPbI_3_-based PSC with a PCBM/ZnO bilayer ETL, its device parameters (*J*_sc_, *V*_oc_, FF, and PCE) are dependent on the thickness of PCBM layer as shown in Fig. 6. Both the *J*_sc_ and PCE increase first and then decrease with the increasing thickness of the PCBM part. Meanwhile, the *V*_oc_ and FF first shift down and up, respectively, and then approach constants as the PCBM thickness increases. Moreover, all the device parameters achieve their highest values when the PCBM thickness is 8 nm. Thus, the optimal thickness of PCBM for the CsPbI_3_-based PSC with a PCBM/ZnO bilayer ETL is 8 nm. For the CsPbI_3_-based PSC with a TiO_2_/ZnO bilayer ETL, all device parameters, except for FF, decrease with increasing thickness of the TiO_2_ part. Meanwhile, the FF first decreases and then slightly increases with the increasing thickness of the TiO_2_ part. During the experiment, the insertion layers between the perovskite and ETL are typically ultra-thin. Hence, the optimal thickness of the TiO_2_ layer for the CsPbI_3_-based PSC with a TiO_2_/ZnO bilayer ETL may be 1 nm.

**Fig. 7:**
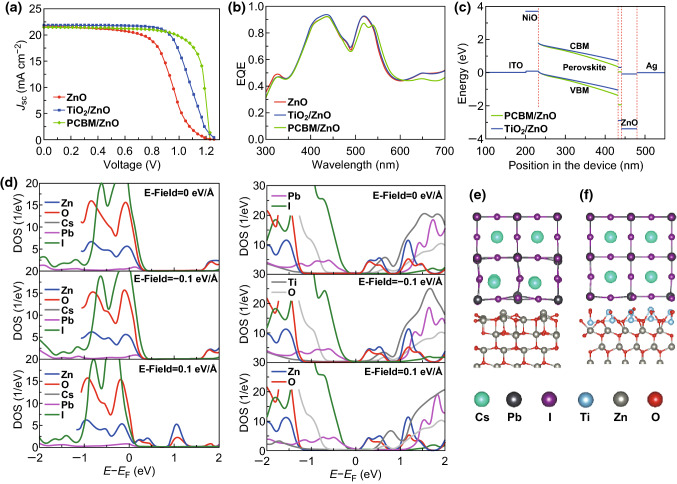
**a** *J*–*V* characteristics of PSCs with a ZnO single-layer ETL, PCBM/ZnO bilayer ETL, and TiO_2_/ZnO bilayer ETL under a simulated AM 1.5 G illumination of 100 mW cm^−2^, in which the doping concentration of the ZnO layer is 10^20^ cm^−3^. **b** EQE spectra of devices based on a 200-nm perovskite layer with different ETLs. **c** Energy band diagrams of the device with a PCBM/ZnO bilayer. **d** DOS of the CsPbI_3_/ZnO and CsPbI_3_/TiO_2_/ZnO interfaces under different electronic fields. The optimized structures of **e** CsPbI_3_/ZnO and **f** CsPbI_3_/TiO_2_/ZnO interfaces are shown

Table 2 lists the PCEs of the CsPbI_3_-based PSCs with a ZnO single-layer ETL, PCBM/ZnO bilayer ETL, and TiO_2_/ZnO bilayer ETL. The detailed calculation parameters are listed in Table S1. Evidently, the PCEs of the CsPbI_3_-based PSCs with a single-layer ZnO ETL can be enlarged by using a bilayer ETL substituting for the single-layer ETL. Under a doping concentration of approximately 10^20^ cm^−3^, the highest PCEs of the CsPbI_3_-based PSCs with a PCBM/ZnO bilayer ETL and TiO_2_/ZnO bilayer ETL are 19.87% and 18.64%, respectively, which are greater than that of the CsPbI_3_-based PSC with a single-layer ZnO ETL (approximately 15.09%). Although these values deviate from the corresponding experimental values, such variation characteristics are consistent with the experimental results [36, 37]. Because the structural variation of the ETL shows a slight effect on the EQE, it further weakens the current trailing in the *J*–*V* curve and then enhances the FF, as shown in Fig. 7a, b and Table 2. However, the increment of the FF of the CsPbI_3_-based PSC with a TiO_2_/ZnO bilayer ETL is inconspicuous such that the PCE of the CsPbI_3_-based PSC with a TiO_2_/ZnO bilayer ETL is lower than that with a PCBM/ZnO bilayer ETL. Notably, the current trailing is not only related to the conductivity of the ETL but also the ion migration and phase separation. Taking the PSC with a TiO_2_/ZnO bilayer ETL as an example, the orbital contributions to the CBM (Zn-*d* and O-*p* orbitals) and VBM (Pb-*p* and I-*p* orbitals) of the CsPbI_3_/TiO_2_/ZnO interfaces are unchanged by small external negative (− 0.1 eV Å^−1^) and positive (0.1 eV Å^−1^) electric fields. Meanwhile, the bandgap is slightly changed by the small external electric field. Such characteristics are different from those of the CsPbI_3_/ZnO interfaces, as shown in Fig. 7d, which suggests weaker ion migration for the CsPbI_3_/TiO_2_/ZnO interface compared to that of the CsPbI_3_/ZnO interface [59]. However, the disordered octahedron of CsPbI_3_ at the CsPbI_3_/ZnO interface region is significantly ordered upon forming the CsPbI_3_/TiO_2_/ZnO interface, relieving the phase separation and interfacial gap states induced by the disordered octahedron of CsPbI_3_ (Fig. 7e, f). In addition, notably, *V*_oc_ is tuned during the process of improved current trailing in the *J*–*V* curve (Fig. 7a). The *V*_oc_ can be decreased and increased to 1.23 and 1.31 V by the TiO_2_/ZnO and PCBM/ZnO bilayer ETL, respectively, as listed in Table 2. The main reason for this is that the additional PCBM film slightly enhances the band bending of the CsPbI_3_ surface at the perovskite/ZnO interface region and then weakens the built-in electric field (viz. the difference between the CBM of CsPbI_3_ and VBM of ZnO as shown in Fig. 7c), while the additional TiO_2_ introduces an opposite effect. From Fig. 7, it can be observed that the TiO_2_/ZnO bilayer ETL slightly improves the *J*_sc_ of the CsPbI_3_-based PSC to 21.83 mA cm^−2^, although it shows a negligible influence on the EQE of the CsPbI_3_-based PSC because the fewer interfacial gap states induced by the TiO_2_/ZnO bilayer ETL can reduce the carrier recombination. In contrast, the PCBM/ZnO bilayer ETL slightly reduces the *J*_sc_ of the CsPbI_3_-based PSC to 21.50 mA cm^−2^ because the corresponding bilayer ETL slightly weakens the EQE and the net carrier generation rate *ΔN*, as shown in Fig. 7b and Table 2.

**Table 2:** Parameters of CsPbI_3_-based PSCs with a ZnO single-layer ETL, PCBM/ZnO bilayer ETL, and TiO_2_/ZnO bilayer ETL

Structure	Doping concentration (cm^−3^)	*ΔN* (cm^−2^ s^−1^)	*J*_sc_ (mA cm^−2^)	*V*_oc_ (V)	FF (%)	PCE (%)
ZnO	10^20^	1.35 × 10^17^	21.60	1.25	55.75	15.09
PCBM/ZnO		1.34 × 10^17^	21.50	1.23	74.99	19.87
TiO_2_/ZnO		1.36 × 10^17^	21.83	1.31	65.34	18.64
ZnO	10^22^	1.35 × 10^17^	21.55	1.25	64.12	17.34
PCBM/ZnO		1.34 × 10^17^	21.48	1.23	75.00	19.87
TiO_2_/ZnO		1.36 × 10^17^	21.79	1.31	74.07	21.06

**Fig. 8:**
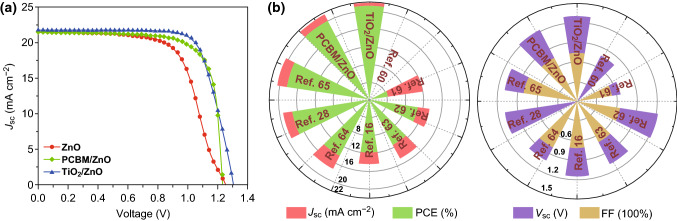
**a** *J*–*V* characteristics of PSCs with a ZnO single-layer ETL, PCBM/ZnO bilayer ETL, and TiO_2_/ZnO bilayer ETL at a doping concentration for the ZnO layer of 10^22^ cm^−3^. **b** Device parameters of PSCs with PCBM/ZnO bilayer ETLs and TiO_2_/ZnO bilayer ETLs optimized by a doping concentration of approximately 10^22^ cm^−3^ in the ZnO layer coupled with the reported device performances of the CsPbI_3_-based PSCs

Notably, although the PCE was significantly improved by employing the bilayer ETL, the FF of the CsPbI_3_-based PSCs with a TiO_2_/ZnO bilayer and the *V*_oc_ of the CsPbI_3_-based PSCs with a PCBM/ZnO bilayer remained lower than expected values. It can be speculated that the PCE can be further improved if the FF and *V*_oc_ are increased. The aforementioned analysis shows that increasing the doping concentration in the ZnO ETL can not only relieve the current trailing in the *J*–*V* curve but also tune the energy of the band level. According to such a mechanism, it can be speculated that the performance of the CsPbI_3_-based PSC with a bilayer ETL can be further improved by increasing the doping concentration of the ZnO layer. The *J*–*V* curves of the CsPbI_3_-based PSCs with a bilayer ETL when the doping concentration of the ZnO layer increases to 10^22^ cm^−3^ are shown in Fig. 8a. Table 2 lists the device parameters of the PSCs with bilayer ETLs and a high doping concentration (approximately 10^22^ cm^−3^). For the PSC with a TiO_2_/ZnO bilayer ETL, its PCE is strongly increased to 21.06% by the increased doping concentration in the ZnO layer because of the enlarged FF of approximately 74.07%. These values are far higher than not only those of the PSC with a TiO_2_/ZnO bilayer ETL and low doping concentration and the PSC with a single-layer ZnO ETL and high doping concentration but also those of previous reports [16, 28, 60–65] as listed in Table 2 and shown in Fig. 8b. As shown in Fig. S6, there is a high charge density at the TiO_2_/ZnO contact, suggesting strong interaction and low contact resistance between the TiO_2_ and ZnO layers. Upon increasing the doping concentration in the ZnO layer, the charge density at the TiO_2_/ZnO contact can be further improved which is beneficial in decreasing the contact resistance between the TiO_2_ and ZnO layers. Meanwhile, because of the strong interlayer interaction at the TiO_2_/ZnO contact, the increment of the doping concentration promotes more carrier transfer to the TiO_2_ buffer layer and improves the conductivity of the TiO_2_/ZnO bilayer ETL. As a result, the FF of the PSC with a TiO_2_/ZnO bilayer ETL increased as the doping concentration in the ZnO layer increased. However, notably, the increased doping concentration in the ZnO layer does not affect the *V*_oc_ and slightly reduces the *J*_sc_ of the PSC with a TiO_2_/ZnO bilayer ETL. Because the band levels of the CsPbI_3_ perovskite and the work function of the electrodes remain unchanged, the increased doping concentration shifts the band levels of the ZnO layer downward, as shown in Fig. S7. In other words, the increment of the positive built-in electric field between the perovskite and ETL equals the increment of the negative built-in electric field between the ETL and electrodes, such that the built-in electric field of the PSC remains unchanged and does not affect the *V*_oc_. In addition, the enlarged doping concentration of the ZnO in the bilayer ETL has a negligible effect on the EQE and net carrier generation rate (Table 2 and Fig. S8), but it can deteriorate the carrier mobility, such that the *J*_sc_ of the PSC with a TiO_2_/ZnO bilayer ETL slightly decreases. These mechanisms are also suitable to the PSC with a PCBM/ZnO bilayer ETL as shown in Figs. S6–S8. Hence, upon increasing the doping concentration of the ZnO layer in the PSC with a PCBM/ZnO bilayer ETL, its variations in *J*_sc_ and *V*_oc_ are same as those of a PSC with a TiO_2_/ZnO bilayer ETL, as listed in Table 2. However, notably, the interlayer interaction between the PCBM and ZnO layer is weak and close to van der Waals interaction, because of low charge density between the PCBM and ZnO layers shown in Fig. S6. Therefore, increasing the doping concentration of the ZnO layer has difficulty improving the charge density between the PCBM and ZnO layers and then enhances the conductivity of the PCBM/ZnO bilayer ETL. Hence, the increased doping concentration does not affect the FF and PCE of the PSC with a PCBM/ZnO bilayer ETL. Moreover, although the improved performances of the PSC with a PCBM/ZnO bilayer ETL are superior to those of other CsPbI_3_-based PSCs [16, 28, 60–65], they are lower than those of the PSC with a TiO_2_/ZnO bilayer ETL, as shown in Fig. 8b. According to the aforementioned analysis, it can be found that modulating the doping concentration of the ZnO layer in the bilayer ETL is an effective means to improve the performance of the PSC with a bilayer ETL when the buffer layer strongly interacts with the ZnO ETL.

## Conclusions

Using device simulations coupled with first-principle calculations, doping engineering of the ZnO ETL and CsPbI_3_/ZnO interface engineering by inserting additional PCBM and TiO_2_ buffer layers are employed to improve the performances of the CsPbI_3_-based PSCs. The results demonstrate that increasing the doping concentration of the ZnO layer alone can relieve the current trailing and introduce the opposite built-in electric field at the ZnO/electrode contact, increasing the FF and decreasing the *V*_oc_. Meanwhile, separately inserting a TiO_2_ buffer layer can reduce the band bending and disordered structure of CsPbI_3_, increasing the *V*_oc_ and PCE. Interestingly, combining these two methods can improve the *V*_oc_, FF, and PCE of the CsPbI_3_-based PSC to 1.31 V, 74.07%, and 21.06%, respectively, because the doping concentration of the ZnO layer in the TiO_2_/ZnO bilayer ETL does not affect the band bending but strongly tunes the conductivity of the TiO_2_/ZnO bilayer ETL. However, the performances of the CsPbI_3_-based PSC with a PCBM/ZnO bilayer ETL are irrespective of the doping concentration in the ZnO layer because the weak interlayer interaction between the PCBM and ZnO layers impedes the effect of the doping concentration in the ZnO layer on the PCBM layer. In addition, these variations are elucidated by the band alignment, density of states, and octahedron order of the corresponding CsPbI_3_/ZnO interfaces. These results provide a comprehensive understanding of the CsPbI_3_/ZnO interface and suggest a guideline to design a high-performance PSC.

## Electronic supplementary material

Below is the link to the electronic supplementary material.

Supplementary material 1 (PDF 596 kb)
