# Supplementary material for: Optimizing the Performance of CsPbI3-Based Perovskite Solar Cells via Doping a ZnO Electron Transport Layer Coupled with Interface Engineering
Source: Nanomicro Lett. 2019 Oct 18;11:91. doi: 10.1007/s40820-019-0320-y (PMC7770773; doi:10.1007/s40820-019-0320-y)
Supplement: Supplementary file 1 — Supplementary material 1 (PDF 596 kb) [file 40820_2019_320_MOESM1_ESM.pdf]

Supporting Information for

Optimizing the Performance of $CsPbI_3$-based Perovskite Solar Cells via Doping a ZnO Electron Transport Layer Coupled with Interface Engineering

Man Yue[1, #], Jie Su[1, #, *], Peng Zhao[1], Zhenhua Lin[1], Jincheng Zhang[1], Jingjing Chang[1, *], Yue Hao[1]

[1]State Key Discipline Laboratory of Wide Band Gap Semiconductor Technology, Shaanxi Joint Key Laboratory of Graphene, School of Microelectronics, Xidian University, 2 South Taibai Road, Xi'an, 710071, People's Republic of China

[#]These authors contributed equally to this work

*Corresponding authors. E-mail: sujie@xidian.edu.cn (Jie Su); jjingchang@xidian.edu.cn (Jingjing Chang)

Supplementary Figures and Table

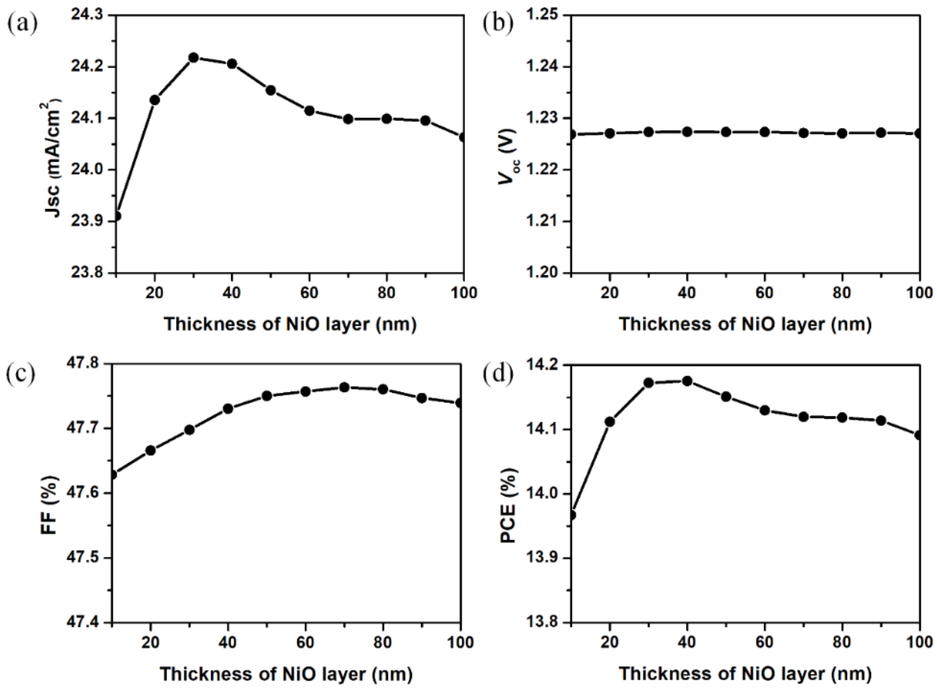

Fig. S1 Device parameters as a function of the thickness of NiO layer

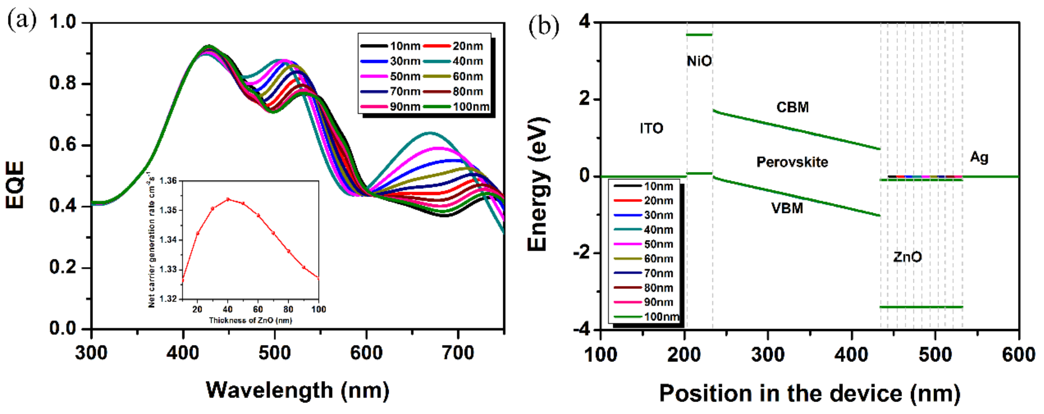

Fig. S2 **a** EQE spectra coupling with the net carrier generation rate, and **b** energy band diagrams of $CsPbI_3$ based PSCs with single-layer ZnO ETL as a function of the thickness of ZnO layer

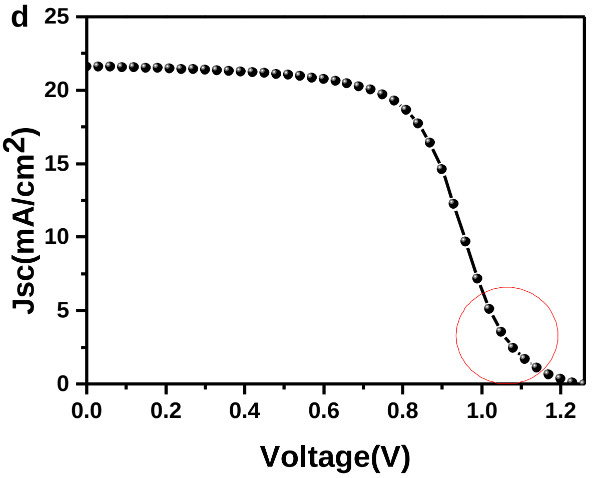

Fig. S3 $J-V$ curve of $CsPbI_3$ based PSC with single-layer ZnO ETL

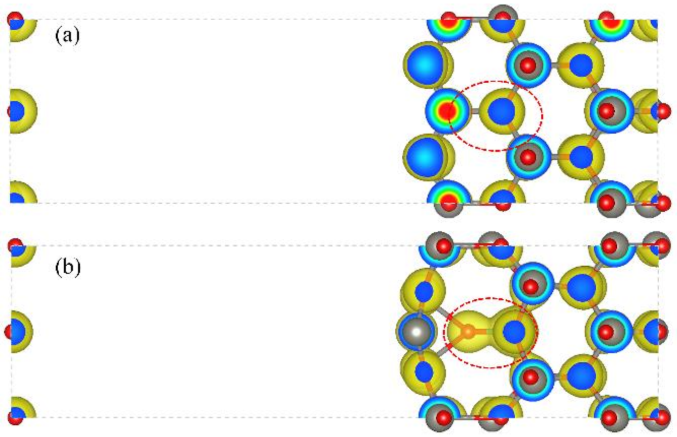

Fig. S4 Charge density of ZnO surfaces **a** without any dopant and **b** with 6.37×10^{21} cm^{-3} Nb-dopant

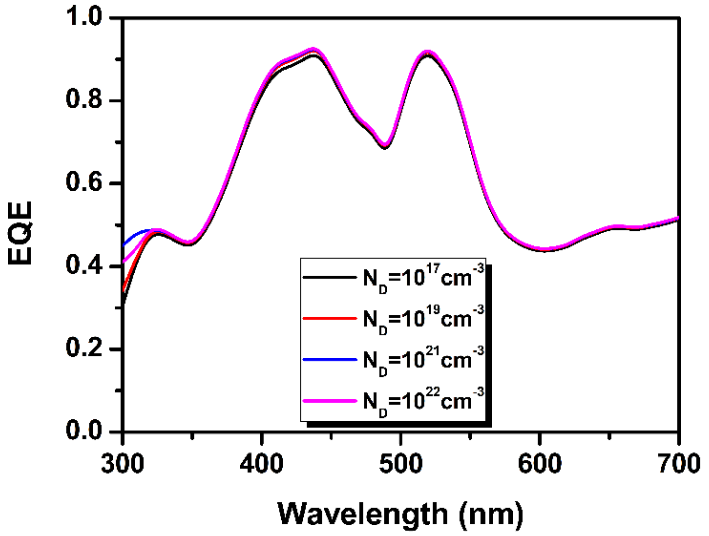

Fig. S5 EQE spectra of $CsPbI_3$ based PSCs with single-layer ZnO ETL dependents on the doping concentration of ZnO layer

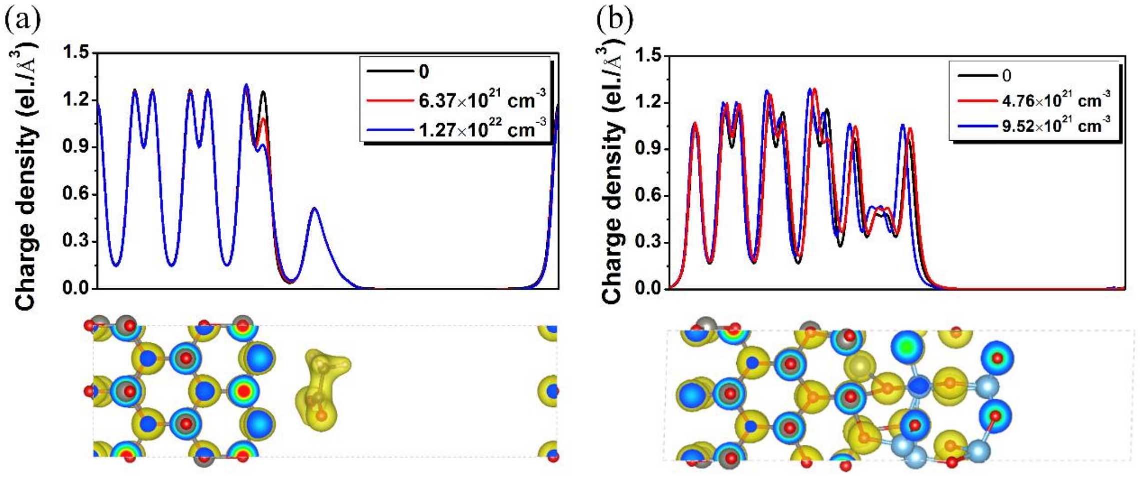

Fig. S6 Charge densities of **a** CH_3COOH/ZnO and **b** TiO_2/ZnO interfaces with different Nb-doping concentrations in the ZnO layers. The up panels are the plane average charge densities along z direction. The down panels are the side view of chage densities of interfaces.

In order to reveal the effect of doping concentration of ZnO layer on the PCBM/ZnO bilayer ETL, the PCBM/ZnO interface should be constructed. However, it should be noted that a proper PCBM/ZnO interface model contains at least 200 atoms due to the large PCBM crystal. Such structure was difficult to be calculated by density functional theory. Hence, the acetic acid (CH_3COOH) was employed to equal with the PCBM according to the structural feature of PCBM. Hence, the CH_3COOH/ZnO interface was constructed. In addition, to investigate the influence the doping concentration on the PCBM/ZnO interface, Nb dopant with different concentration was introduced into the ZnO layer of these interfaces. It could

be found that the charge densities between PCBM and ZnO layers was low, which suggesting weak interlayer interactions at CH_3COOH/ZnO interface which was close to the van der waals interactions. Moreover, the increased Nb-doping concentration in ZnO layer has tiny effects on the low charge densities, which directly determined the conductivity, between the ZnO and CH_3COOH layers. Hence, the increased doping concentration in ZnO layer unaffected the conductivity of CH_3COOH/ZnO interface. In other words, the increased doping concentration in ZnO layer could not improve the conductivity of PCBM/ZnO interface.

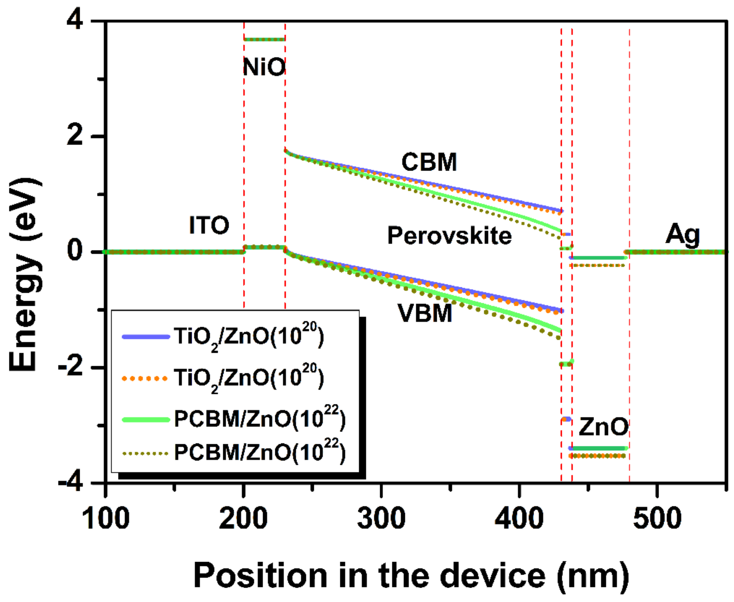

Fig. S7 Energy band diagrams of $CsPbI_3$ based PSCs with bilayer ETLs as a function of the doping concentration in ZnO layer

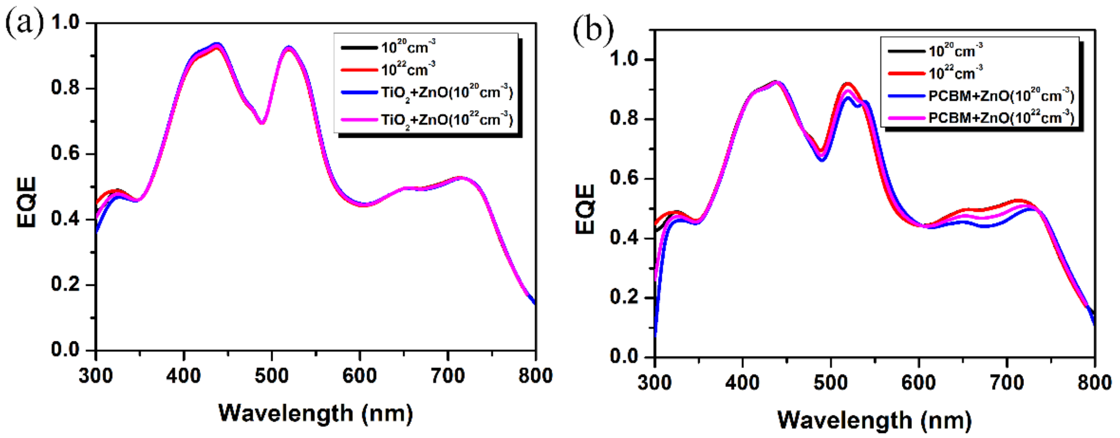

Fig. S8 EQE spectra of $CsPbI_3$ based PSCs with single-layer ZnO ETL, bilayerPCBM/ZnO ETL, and bilayerTiO_2/ZnO ETL

Table S1 The detailed simulation parameters of $CsPbI_3$ based PSCs with optimized ZnO single-layer ETL, PCBM/ZnO bilayer ETL, and TiO_2/ZnO bilayer ETL

Parameters	NiO	$CsPbI_3$	PCBM	ZnO	TiO_2
Thickness (nm)	30	200	8	40	1
ε_r	12	6	4	9	100
E_g (eV)	3.6	1.73	2	3.3	3.2
λ (eV)	1.7	3.6	3.9	4.4	4
N_C (cm^{-3})	2.5×10^{20}	1.49×10^{18}	1×10^{21}	2.2×10^{18}	1×10^{21}
N_V (cm^{-3})	2.5×10^{20}	2.2×10^{18}	2×10^{20}	1.8×10^{19}	2×10^{20}
N_A (cm^{-3})	1×10^{16}	-	-	-	-
N_D (cm^{-3})	-		1×10^{20}	1×10^{20}	1×10^{20}
μ_n (cm^2/Vs)	0.01	25	0.01	100	0.006
μ_p (cm^2/Vs)	0.01	25	0.01	25	0.006
